# Supplementary material for: Top-down regulation of ingestive behavior fragmentation
Source: bioRxiv. 2026 Apr 3:2026.03.31.715709. Preprint. [Version 1] doi: 10.64898/2026.03.31.715709 (PMC13060137; doi:10.64898/2026.03.31.715709)
Supplement: Supplement 1 [file NIHPP2026.03.31.715709v1-supplement-1.pdf]

bioRxiv preprint doi: https://doi.org/10.64898/2026.03.31.715709; this version posted April 3, 2026. The copyright holder for this preprint (which was not certified by peer review) is the author/funder, who has granted bioRxiv a license to display the preprint in perpetuity. It is made available under aCC-BY 4.0 International license.

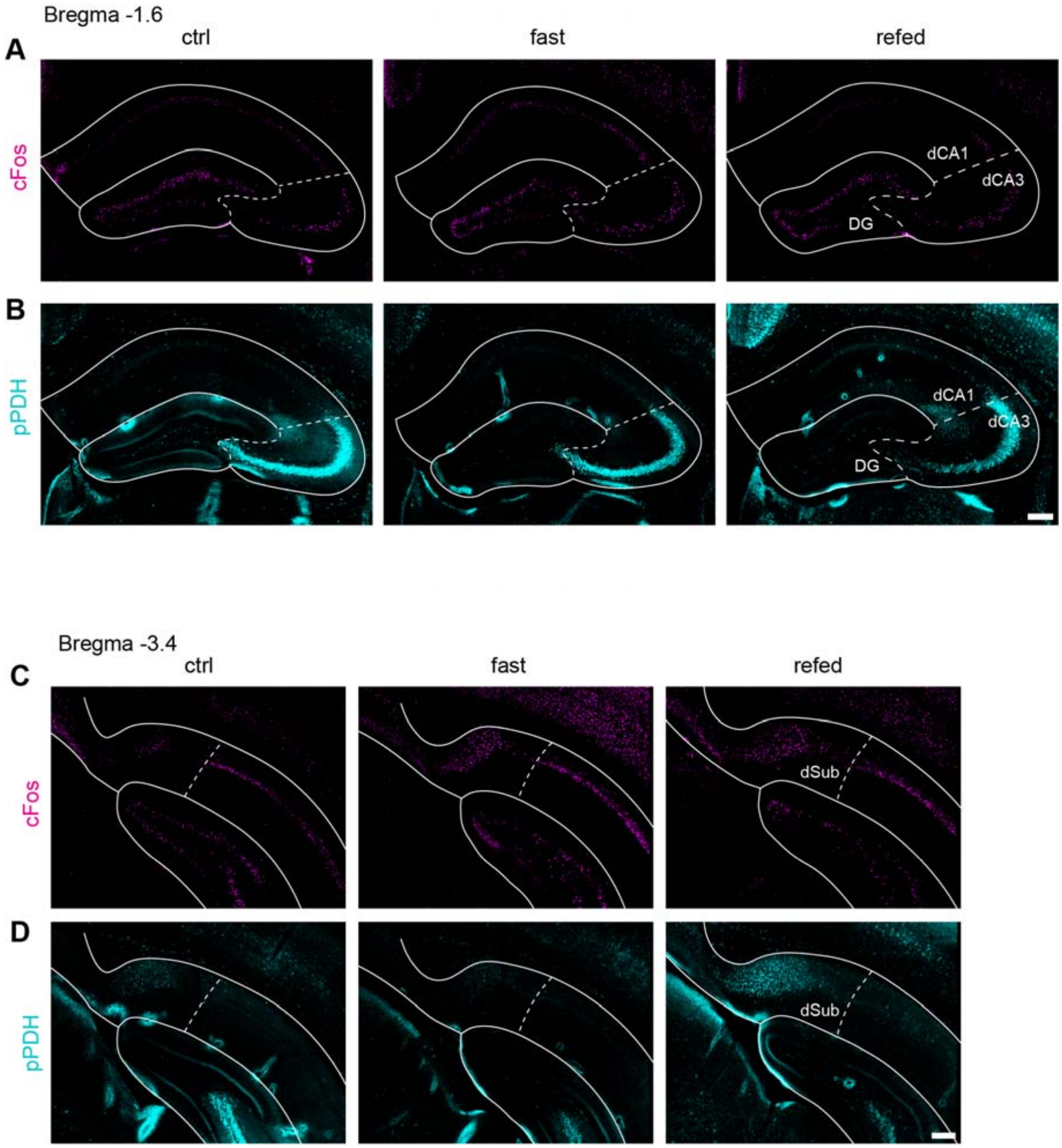

Fig. S1. Bidirectional activity mapping of the dorsal hippocampus.

(A) Representative images of cFos staining in DG, CA1 and CA3.

(B) Representative images of pPDH staining in DG, CA1 and CA3.

(C) Representative images of cFos staining in posterior part of the dorsal hippocampus, showing dSub.

(D) Representative images of pPDH staining in posterior part of the dorsal hippocampus, showing dSub.

bioRxiv preprint doi: https://doi.org/10.64898/2026.03.31.715709; this version posted April 3, 2026. The copyright holder for this preprint (which was not certified by peer review) is the author/funder, who has granted bioRxiv a license to display the preprint in perpetuity. It is made available under aCC-BY 4.0 International license.

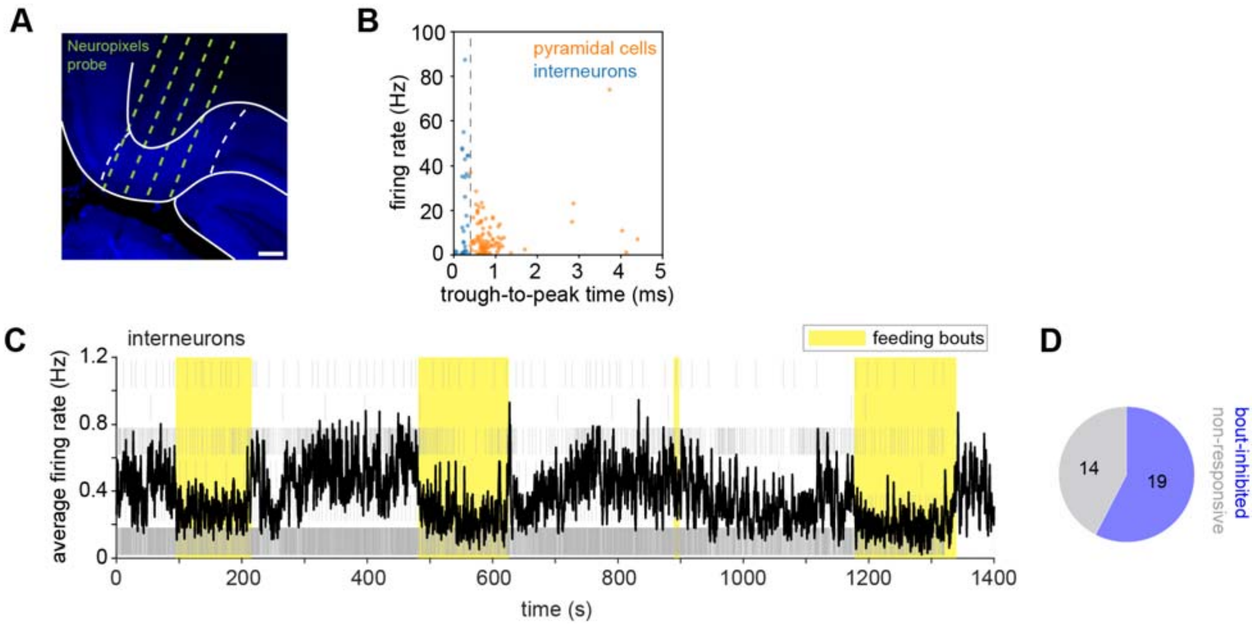

Fig. S2. Additional characterization of dSub Neuropixels 2.0 recording.
(A) Representative histology showing Neuropixels 2.0 probe implant. Scale bar is 200 μm.
(B) Criteria of classification of pyramidal neurons and interneurons. Sorted units with trough-to-peak time (TTP) < 0.4 ms are assigned to interneurons, and units with TTP > 0.4 ms are assigned to pyramidal neurons.
(C) Raster plot and populational average firing rate of all interneurons from a representative mouse. For readability given the long time window, only every 20th spike is displayed in the raster plot.
(D) Proportion of bout-inhibited and non-responsive interneurons. Total of 33 neurons from 4 mice.

bioRxiv preprint doi: https://doi.org/10.64898/2026.03.31.715709; this version posted April 3, 2026. The copyright holder for this preprint (which was not certified by peer review) is the author/funder, who has granted bioRxiv a license to display the preprint in perpetuity. It is made available under aCC-BY 4.0 International license.

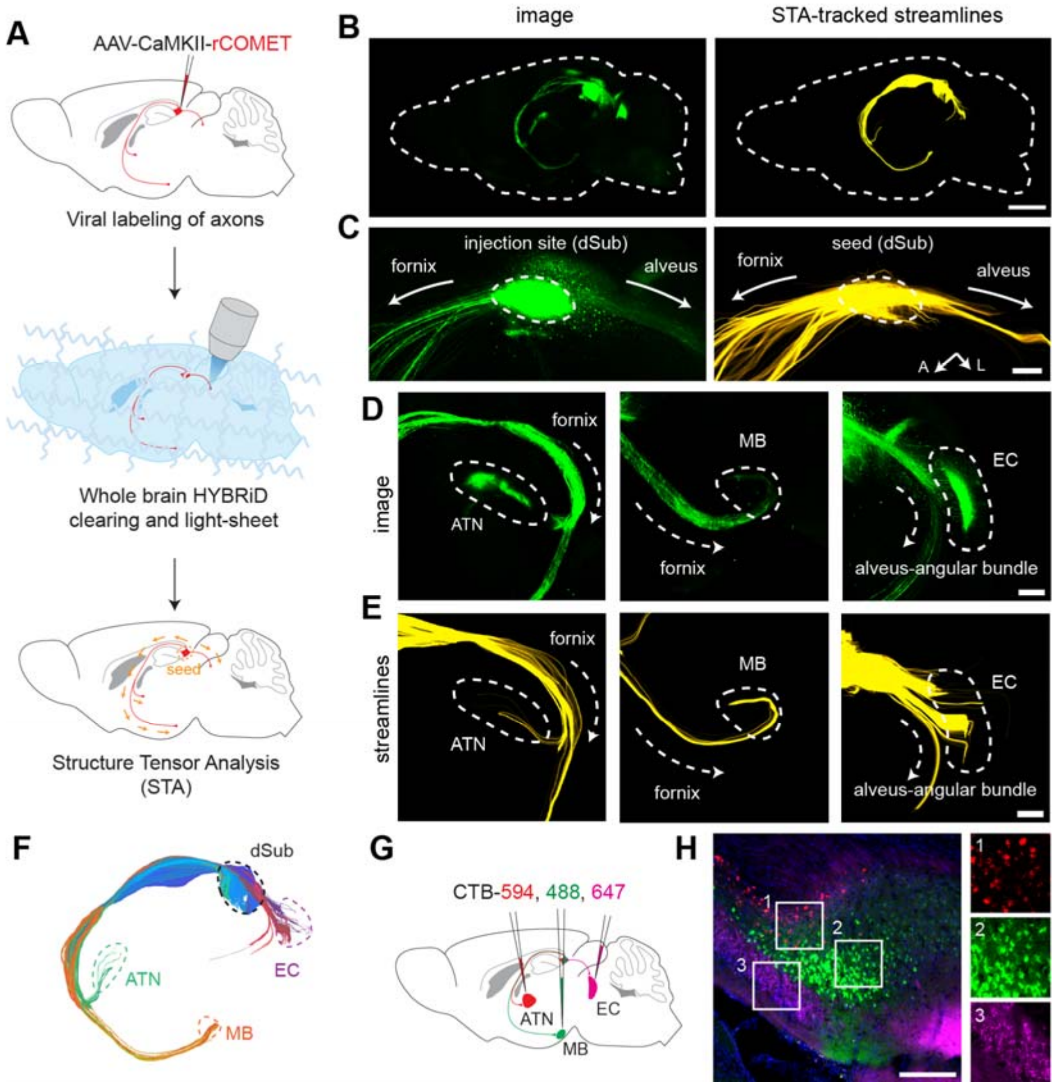

Fig. S3. Mapping of extrahippocampal projections from dSub.

(A) Schematic of virus injection, tissue clearing and light-sheet imaging, and structure tensor analysis.

(B) Representative whole-brain image and reconstructed streamlines (direction of greatest tract diffusion).

(C) Representative image and reconstructed streamlines around the dSub, showing two main streams of projections (fornix and alveus).

(D-E) Representative images and reconstructed streamlines around ATN, MB and EC.

(F) Reconstructed streamlines from dSub, colored by projection terminals.

(G) Schematic of 3-color CTB retrograde tracing from ATN, MB and EC.

(H) Representative images of dSub, showing distinct soma localizations of cells projecting to different targets.

Scale bars are 1 mm for (B), and 200 μm for (C-E) and (H).

bioRxiv preprint doi: https://doi.org/10.64898/2026.03.31.715709; this version posted April 3, 2026. The copyright holder for this preprint (which was not certified by peer review) is the author/funder, who has granted bioRxiv a license to display the preprint in perpetuity. It is made available under aCC-BY 4.0 International license.

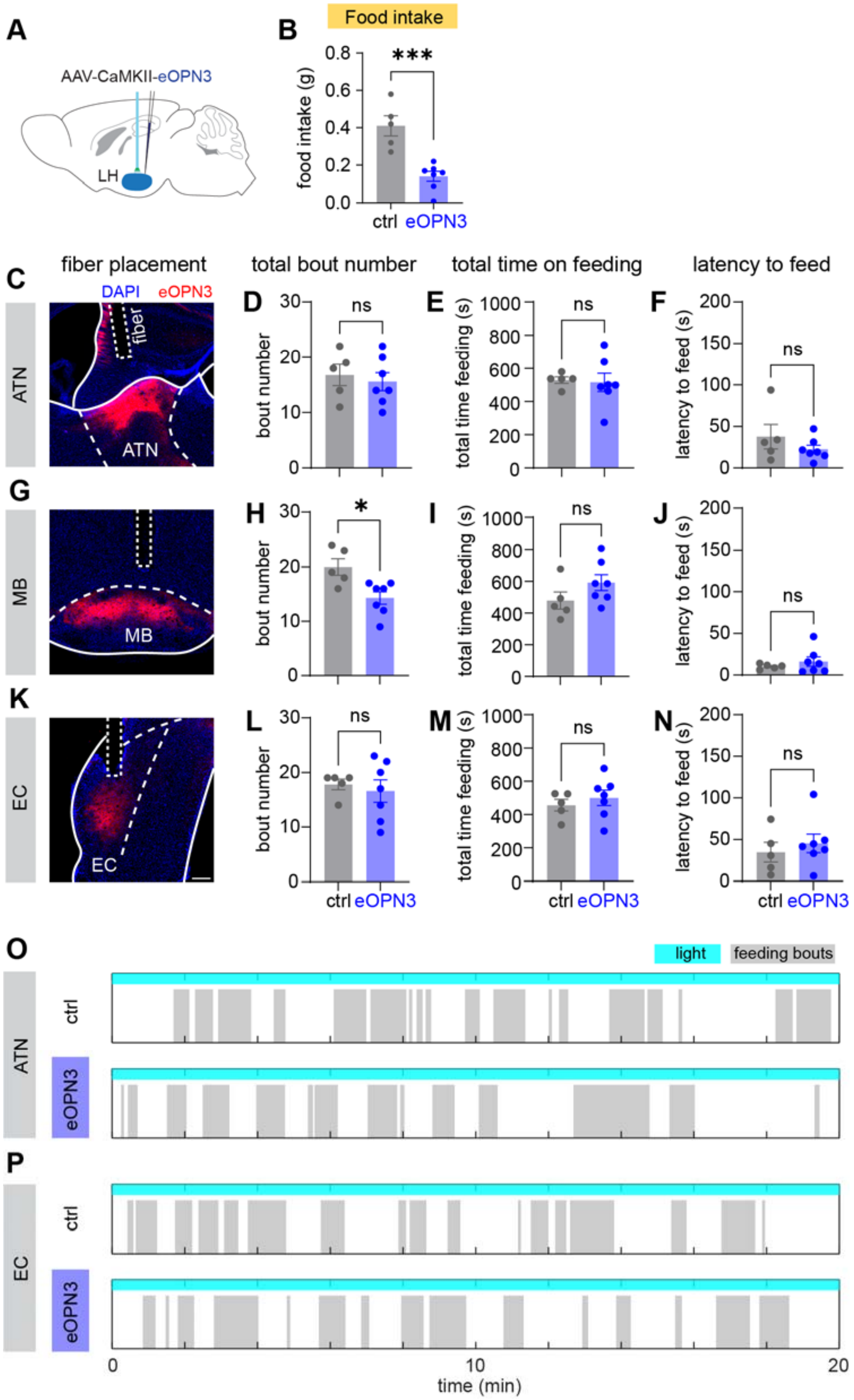

Fig. S4. Additional characterization of feeding microstructure of dSub projection-specific photoinhibition.

(A) Schematic of virus injection and fiber implantation for LH photoinhibition experiment. N = 5 mice for ctrl group and N = 7 mice for eOPN3 group.

(B) Food intake analysis in the LH photoinhibition experiment.

bioRxiv preprint doi: https://doi.org/10.64898/2026.03.31.715709; this version posted April 3, 2026. The copyright holder for this preprint (which was not certified by peer review) is the author/funder, who has granted bioRxiv a license to display the preprint in perpetuity. It is made available under aCC-BY 4.0 International license.

(C) Representative histology of ATN of dSub-ATN eOPN3 cohort, showing fiber placement and expression of eOPN3 in axons in ATN.
(D) Total number of feeding bouts under dSub-ATN photoinhibition.
(E) Total time spent in feeding bouts under dSub-ATN photoinhibition.
(F) Latency to the first feeding bout under dSub-ATN photoinhibition.
(G-J) Representative histology and quantifications for dSub-MB photoinhibition experiment.
(K-N) Representative histology and quantifications for dSub-EC photoinhibition experiment.
(O-P) Representative raster of feeding bouts of a ctrl mouse and an eOPN3 mouse of dSub-ATN and dSub-EC photoinhibition.
All values are mean ± SEM. Statistics determined by Student's t-test for mean duration and food intake analyses, and two-tailed permutation KS test for ECDF analyses. $*p < 0.05$; $***p < 0.001$. All scale bars are 200 μm.

bioRxiv preprint doi: https://doi.org/10.64898/2026.03.31.715709; this version posted April 3, 2026. The copyright holder for this preprint (which was not certified by peer review) is the author/funder, who has granted bioRxiv a license to display the preprint in perpetuity. It is made available under aCC-BY 4.0 International license.

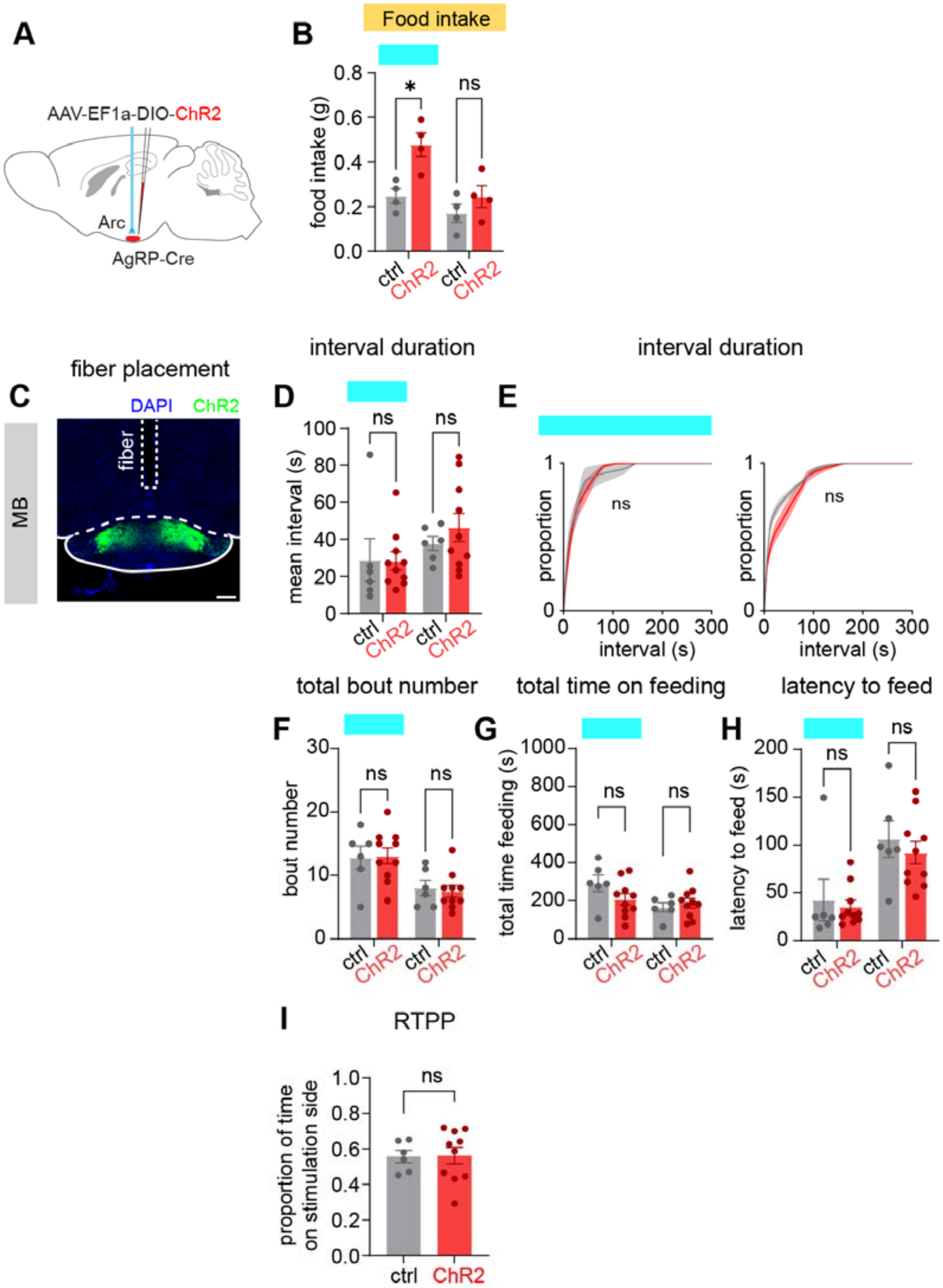

Fig. S5. Additional characterization of feeding microstructure of dSub-MB photo activation.

(A) Schematic of virus injection and fiber implantation for Arc AgRP photoactivation experiments.

(B) Total food intake amount under Arc AgRP photoactivation.

(C) Representative histology of MB from dSub-MB ChR2 cohort, showing fiber placement and expression of ChR2 in axons in MB.

(D-E) Mean and ECDF of interval duration in light ON phase and light OFF phase of dSub-MB photoactivation.

bioRxiv preprint doi: https://doi.org/10.64898/2026.03.31.715709; this version posted April 3, 2026. The copyright holder for this preprint (which was not certified by peer review) is the author/funder, who has granted bioRxiv a license to display the preprint in perpetuity. It is made available under aCC-BY 4.0 International license.

(F-H) Total feeding bout count, total time spent in feeding bouts, and latency to the first feeding bout under dSub-MB photoactivation.

(I) Time spent on the stimulation side in a real-time place preference (RTPP) experiment.

All values are mean ± SEM. Statistics determined by Student's t-test for mean duration and food intake analyses, and two-tailed permutation KS test for ECDF analyses. $*p < 0.05$. Scale bar is 200 μm.

bioRxiv preprint doi: https://doi.org/10.64898/2026.03.31.715709; this version posted April 3, 2026. The copyright holder for this preprint (which was not certified by peer review) is the author/funder, who has granted bioRxiv a license to display the preprint in perpetuity. It is made available under aCC-BY 4.0 International license.

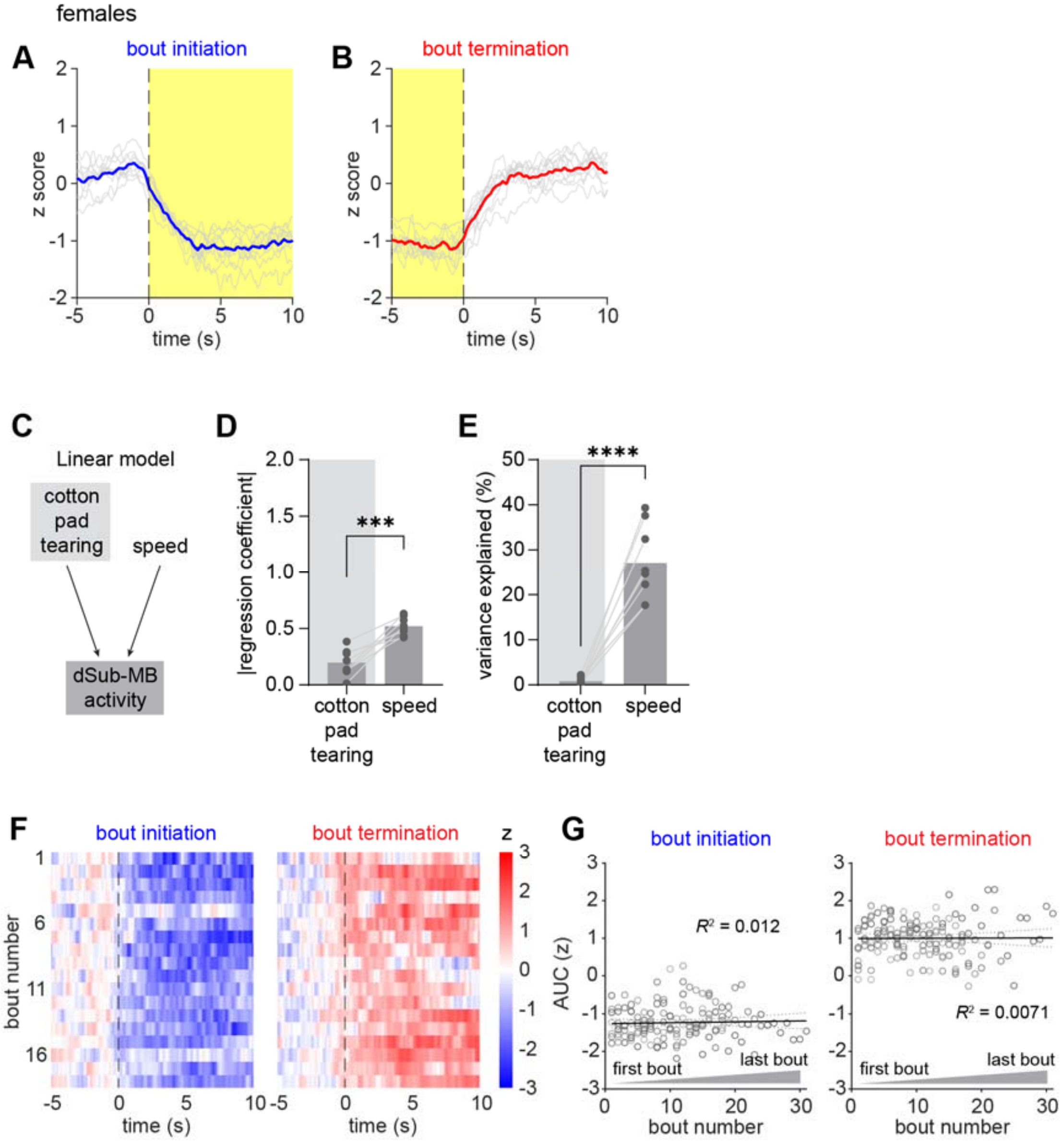

Fig. S6. Calcium response of dSub-MB circuit during feeding and movement-related behaviors.

(A-B) Averaged calcium signal in female mice triggered on initiation and termination of feeding bouts. N = 10 mice.

(C) Schematic of the linear regression of dSub-MB calcium activity against bouts of cotton pad tearing and speed of locomotion.

(D-E) Absolute value of regression coefficient and percentage of variance explained of dSub-MB activity by cotton pad tearing and speed.

(F) Representative heatmaps showing dSub-MB activity triggered on initiation and termination of feeding for all bouts from a given mouse.

(G) Correlation between AUC of neural activity during a feeding bout and the bout number within a session.

Statistics determined by paired t tests in (D-E) and mixed-effect linear regression in (G).

bioRxiv preprint doi: https://doi.org/10.64898/2026.03.31.715709; this version posted April 3, 2026. The copyright holder for this preprint (which was not certified by peer review) is the author/funder, who has granted bioRxiv a license to display the preprint in perpetuity. It is made available under aCC-BY 4.0 International license.

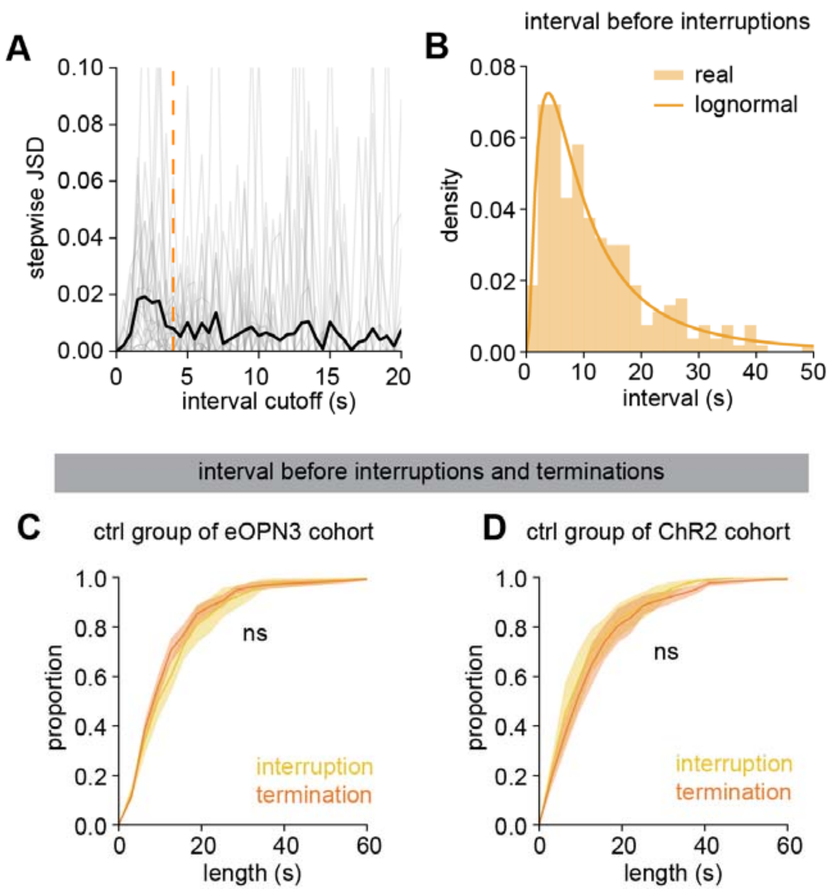

Fig. S7. Additional characterizations of the interruptions.

(A) Selection of a threshold to distinguish feeding bout interruptions from feeding bout terminations, and its effect on the distribution of feeding bout durations. Y axis shows difference in the empirical distribution of bout durations given a cutoff of t vs t + 0.5s, quantified by the Jensen–Shannon divergence (JSD). Orange dashed line shows the used cutoff at 4 s. Gray traces show individual mice, and black shows the average across all mice. N = 12 mice, combining all ctrl mice from the eOPN3 and ChR2 cohorts.

(B) Histogram of the intervals between each intra-bout interruption or bout termination and the preceding intra-bout interruption (or bout start) in the experimental data (bars), and the fitted lognormal distribution used to generate interruption input pulses in the model. Data from the ctrl groups of the eOPN3 and the ChR2 cohorts.

(C) ECDF of the interval between each intra-bout interruption or bout termination and the preceding intra-bout interruption (or bout start) for the ctrl group in the eOPN3 cohort.

(D) ECDF of the interval between each intra-bout interruption or bout termination and the preceding intra-bout interruption (or bout start) for the ctrl group in the ChR2 cohort.

Statistics determined by two-tailed permutation KS tests in (C-D).

bioRxiv preprint doi: https://doi.org/10.64898/2026.03.31.715709; this version posted April 3, 2026. The copyright holder for this preprint (which was not certified by peer review) is the author/funder, who has granted bioRxiv a license to display the preprint in perpetuity. It is made available under aCC-BY 4.0 International license.

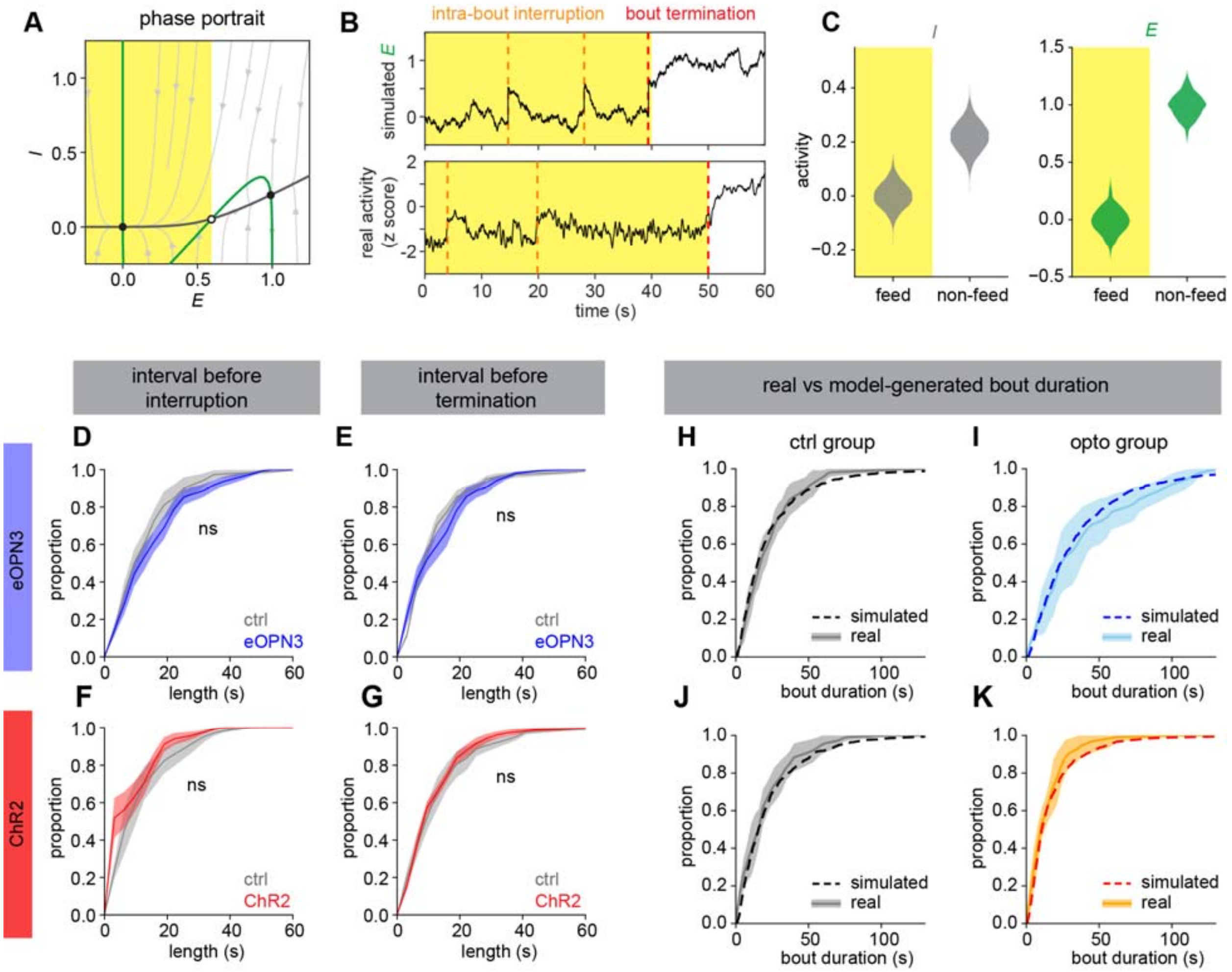

Fig. S8. Additional characterizations of the model.

(A) Phase portrait of the model. Nullclines of E and I populations are shown by the green and black lines, respectively, and flow field are shown by the light gray arrows. Solid dots are stable fixed points, hollow dots are unstable fixed points. We use the E population state at the unstable fixed point as the approximate threshold between states; sub-threshold E population values correspond to the feeding state, super-threshold to the non-feeding state.

(B) Simulated activity of the E population (above) compared to fiber photometry recording of dSub-MB projection neurons from a representative mouse (below).

(C) Activity distribution of the model I population and E population in the feeding and nonfeeding states.

(D) ECDF of the interval between each intra-bout interruption and the preceding intra-bout interruption (or bout start) for the ctrl group in the dSub-MB photoinhibition cohort.

(E) ECDF of intervals preceding a termination for the ctrl group and eOPN3 group in the dSub-MB photoinhibition cohort.

(F-G) Same ECDFs for the ctrl group and ChR2 group in the dSub-MB photoactivation cohort.

(H-I) ECDF of bout durations in real data and model-simulated data, for the eOPN3 cohort.

(J-K) ECDF of bout duration from real data and model-simulated data, of the ChR2 cohort.

bioRxiv preprint doi: https://doi.org/10.64898/2026.03.31.715709; this version posted April 3, 2026. The copyright holder for this preprint (which was not certified by peer review) is the author/funder, who has granted bioRxiv a license to display the preprint in perpetuity. It is made available under aCC-BY 4.0 International license.

Values are mean ± SEM for (D-G) and mean ± SD for (H-K). Statistics determined by two-tailed permutation KS tests for (D-G).

bioRxiv preprint doi: https://doi.org/10.64898/2026.03.31.715709; this version posted April 3, 2026. The copyright holder for this preprint (which was not certified by peer review) is the author/funder, who has granted bioRxiv a license to display the preprint in perpetuity. It is made available under aCC-BY 4.0 International license.

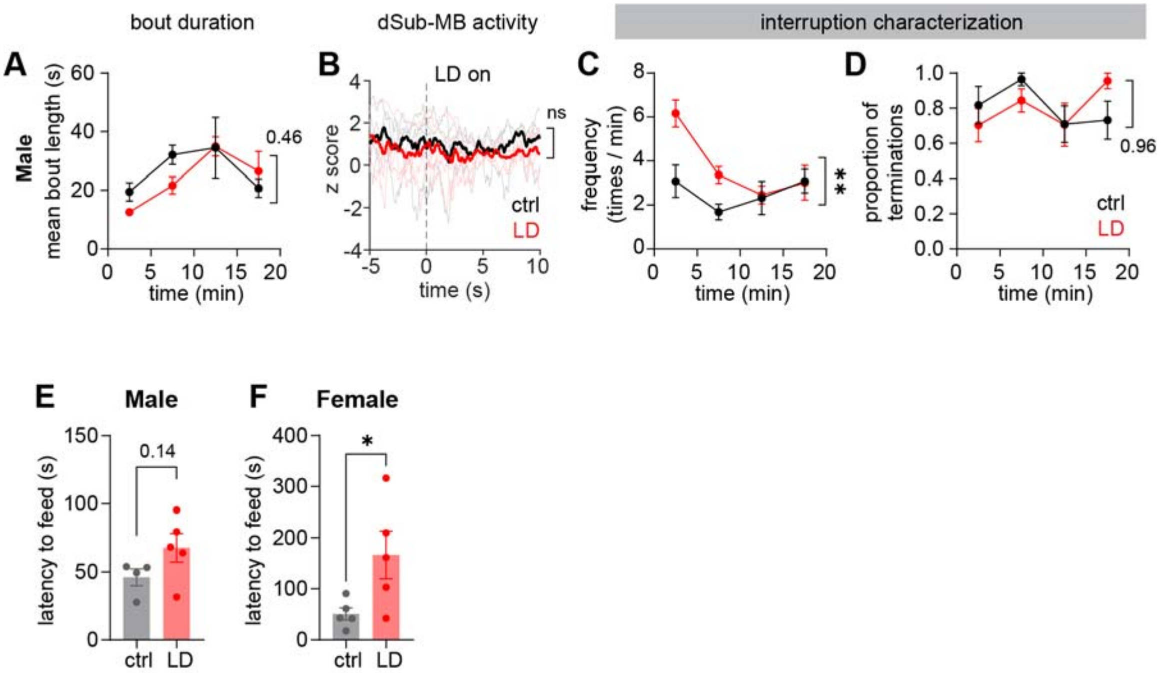

Fig. S9. Additional characterizations of looming disc experiment.
(A) Time course of the bout duration in male mice. N = 4 mice for ctrl group and N = 5 mice for LD group.
(B) Calcium activity of dSub-MB in female mice triggered on the first appearance of the looming disk.
(C-D) Time course of the total frequency of interruptions and terminations, and percentage of terminations, in male mice.
(E-F) Latency to the first feeding bout in male and female mice, in control conditions vs in the presence of a looming disc (LD).
All values are mean ± SEM. Statistics determined by two-way ANOVA (main effect of group) for (A-D), and Student's t tests for (E-F). $*p < 0.05$, $**p < 0.01$. P values for non-significant comparisons are labeled.
